# Dose-Dependent Differences in HIV Inhibition by Different Interferon Alpha Subtypes While Having Overall Similar Biologic Effects

**DOI:** 10.1128/mSphere.00637-18

**Published:** 2019-02-13

**Authors:** Erika Schlaepfer, Audrey Fahrny, Maarja Gruenbach, Stefan P. Kuster, Viviana Simon, Gideon Schreiber, Roberto F. Speck

**Affiliations:** a Department of Infectious Diseases and Hospital Epidemiology, University Hospital of Zurich, Zurich, Switzerland; b Department of Microbiology, The Global Health and Emerging Pathogens Institute, Icahn School of Medicine at Mount Sinai, New York, New York, USA; c Department of Biomolecular Sciences, Weizmann Institute of Science, Rehovot, Israel; University of Michigan Medical School

**Keywords:** antiviral therapy, human immunodeficiency virus, interferons, therapeutic efficacy

## Abstract

Type I interferons (IFNs) are key players in the antiviral immune response. Interferon alpha (IFN-α) belongs to this class of IFNs and comprises 12 subtypes that differ from each other in their binding affinities for a common receptor and, thus, in their signaling potencies. Recent data suggest that IFN-α6 and -α14 are the most potent IFN-α subtypes in restricting HIV replication when applied exogenously. However, in the context of antiviral therapy, IFNs are administered at high doses, which may compensate for differences in potency seen between IFN-α subtypes. In this study, we reexamined whether IFN-α subtypes induce different biological activities, with a focus on how IFN-α treatment dose affects cellular responses to HIV in primary CD4^+^ T cells, peripheral blood mononuclear cells (PBMCs), and macrophages. We found that the subtypes’ antiviral activities were dose dependent, with >90% inhibition of HIV replication at a high dose of all IFN-αs except the weak IFN-α/β receptor (IFNAR) binder, IFN-α1. The quality of the responses engendered by IFN-α1, -α2, -α6, and -α14 was highly comparable, with essentially the same set of genes induced by all four subtypes. Hierarchal cluster analysis revealed that the individual donors were stronger determinants for the IFN-stimulated-gene (ISG) responses than the specific IFN-α subtype used for stimulation. Notably, IFN-α2-derived mutants with substantially reduced IFNAR2 binding still inhibited HIV replication efficiently, whereas mutants with increased IFNAR1 binding potentiated antiviral activity. Overall, our results support the idea that IFN-α subtypes do not induce different biological responses, given that each subtype is exogenously applied at bioequivalent doses.

**IMPORTANCE** Elucidating the functional role of the IFN-α subtypes is of particular importance for the development of efficacious therapies using exogenous IFN-α. Specifically, this will help define whether IFN therapy should be based on the use of pathogen-dependent IFN subtypes or, rather, IFN mutants with optimized IFNAR binding properties.

## Abstract (precis)

Elucidating the functional role of the IFN-α subtypes is of particular importance for the development of efficacious therapies using exogenous IFN-α. Specifically, this will help define whether IFN therapy should be based on the use of pathogen-dependent IFN subtypes or, rather, IFN mutants with optimized IFNAR binding properties.

## INTRODUCTION

Human type I interferons (IFNs) are key players in protection against viral infections, and their antiviral potency has been harnessed for treating chronic hepatitis B virus (HBV) and hepatitis C virus (HCV) infections, as well as in clinical trials for HIV-1-infected individuals (1–4). The family of human interferon alpha (IFN-α) is a subset of type I IFNs comprising 12 distinct proteins that display great genetic and structural homology (75 to 99% amino acid sequence identity) (5). All IFN-α subtypes bind to the ubiquitously expressed type I IFN-α/β receptor (IFNAR), which is composed of two transmembrane subunits, IFNAR1 and IFNAR2. Although the IFN-α subtypes form structurally highly comparable ligand-receptor complexes (6), they differ from one another in their affinities for the IFNAR subunits, ranging from 0.5 to 5 µM for IFNAR1 and 0.4 to 5 nM for IFNAR2, with the exception of IFN-α1, which displays substantially lower affinities (7). Binding to the IFNAR complex triggers, via the Jak/STAT pathway, the transcription of hundreds of IFN-stimulated genes (ISGs) (8). Modulation of IFN-α signaling by an array of other factors and signaling cascades (reviewed in reference 9), as well as cell type-, pathogen-, and microenvironment-specific determinants, results in the induction of distinct subsets of cellular effectors that mediate the diverse biological responses attributed to IFN-α, such as the hundreds of ISGs associated with the establishment of an antiviral state in infected and neighboring cells (10, 11).

The reason for the existence of so many IFN-α subtypes, which all signal through the same receptor, remains a controversial subject. Indeed, distinct IFN-α subtype profiles are elicited in both pathogen- and cell type-specific manners (12–15), yet whether a distinct profile is necessary for an effective antiviral immune response is still a matter of debate (14, 16–18). One perception is that the IFN-α subtypes exert distinct functional roles; another perception is that not all the IFN-α subtypes are essential in a cell, but the built-in redundancy is beneficial in case of failure of certain subtypes due to viral evasion strategies (19). As each IFN-α subtype is under the control of its own promoter, the presence of many subtypes allows dynamics in production levels (15, 20). Notably, all we know about IFN subtypes’ biological significance is based on their exogenous administration in various *in vitro* or *in vivo* settings (1, 16, 19, 21–24).

We previously demonstrated that, although all IFN-α subtypes trigger an antiviral state (6), the affinity and stability of the ternary IFN-α–receptor complex dictate, to a large extent, the distinct biological activities (25). Notably, the high-affinity subtypes human IFN-α5, -α6, -α8, and -α14 have been shown to be the most potent in inhibiting HIV replication *in vitro* and in humanized mice (16, 17, 26). Moreover, studies assessing the antiviral potency of murine IFN-α transgene therapy in mouse models of infection with influenza virus, murine cytomegalovirus, and herpes simplex virus revealed that IFN-α subtypes exerted distinct antiviral potencies in a virus-specific manner (21–24). These results suggest that individual IFN-α subtypes possess distinct qualitative properties. Nevertheless, in these studies, IFN subtypes were provided either as transfected transgenes or at a single dose, thus precluding the identification of possible dose-dependent effects and a distinction between potency and therapeutic effectiveness. In addition, antiviral potencies were not always consistent across related experimental investigations (14, 21, 22, 24). On the other hand, others have shown that given sufficiently high type I IFN doses, the induced ISG responses are qualitatively and quantitatively similar between IFN subtypes (14, 27). In this regard, as the induced ISG profile ultimately dictates the cellular response to IFN signaling, it is difficult to resolve how IFN-α subtypes could induce functionally divergent activities. Moreover, when interferons are administered at pharmacologic (i.e., supraphysiological) doses (19), it is likely that each IFN-α subtype could induce its maximum effect, compensating for differences in potency.

The prevailing ambiguity regarding the IFN-α subtypes’ functional role (reviewed in reference 28) spurred us to reexamine whether these 12 highly similar cytokines, which all trigger the same JAK/STAT signaling pathway, can genuinely induce differential activities or whether our understanding of the IFN-α subtypes’ intrinsic properties has been masked by dose-related effects. To this end, we investigated the antiviral properties of IFN-α subtypes in *ex vivo* HIV-infected primary cells with a focus on assessing the dose-dependent effects of a selected set of IFN-α subtypes. Furthermore, using a panel of IFN-α mutants with different affinities for IFNAR1 and IFNAR2, we defined dominant signaling domains responsible for anti-HIV activity. Elucidating the functional role of the IFN-α subtypes is of particular importance for the development of efficacious therapies using exogenous IFN-α, in that such findings will clarify whether IFN therapy should be based on pathogen-dependent subtype selection or, rather, on the use of an IFN-α mutant with optimized IFNAR binding properties, or whether the effects observed for any one IFN are only a question of dose.

## RESULTS

### Dose-dependent differences in signaling potency between IFN-α1, -α2, and -α6 with saturation of subtype-specific differences at 1,000 pg/ml.

**FIG 1:**
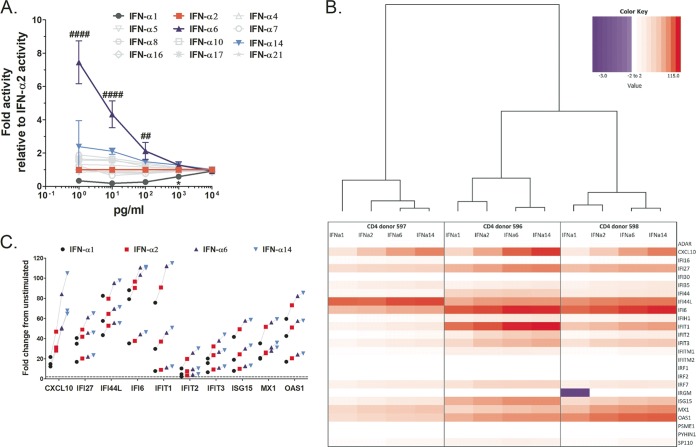
Signaling activities of IFN-α subtypes converge at high treatment doses, which translates into comparable ISG induction profiles between subtypes. (A) Dose-dependent induction of gene expression by all 12 IFN-α subtypes in cells carrying an ISRE-luciferase construct. Significance testing was done for each IFN-α dose using one-way ANOVAs on fold activity relative to the activity of IFN-α2. Symbols for comparisons with IFN-α2 are as follows: *, IFN-α1; #, IFN-α6. Presentation of data is described in “Statistical analysis” in Materials and Methods for all figures. (B) Heat map showing genes induced in primary CD4^+^ T cells from three independent donors stimulated with 1,000 pg/ml of IFN-α1, -α2, -α6, or -α14 compared to their induction in unstimulated controls (i.e., fold upregulation normalized to the expression in the unstimulated control; the values −3 to 115 represent the extremes of down- and upregulation as normalized to the result for the unstimulated control), and hierarchical clustering of the induced-gene-response profiles. For this assay, the interferons and receptors RT^2^ Profile PCR array (Qiagen) was used, but only results for genes of interest (ISGs) are shown; see Fig. S2 in the supplemental material for results for the full array of genes included in the PCR array. (C) Detailed view of genes quantitatively differentially induced by IFN-α1, -α2, -α6, or -α14. Lines connect responses from each donor, and the horizontal dashed line represents 2-fold change in gene expression compared to expression in unstimulated controls. These 10 genes represent the set of genes that showed significantly different expression levels between at least one pair of IFN-α subtypes in repeated measures (RM) two-way ANOVAs of the data set shown in panel B (ANOVA results are summarized in Table S2). Note that all 10 genes were induced >2-fold.

The overarching aim of this study was to elucidate whether exogenously applied IFN-α subtypes engender qualitatively different biological responses or whether discrepancies between subtypes are merely a consequence of the applied dose (i.e., quantitative). As the cellular responses to IFN-α signaling are highly plastic due to signal modulation in a cell type- and context-dependent manner (6, 11), we first screened the 12 distinct IFN-α proteins over a range of doses for their ability to trigger the JAK/STAT pathway and induce reporter gene expression using a reporter cell line transduced with an IFN-stimulated response element (ISRE)-luciferase construct. Upon treatment with IFN-α, these reporter cells are induced to express luciferase, and thus, the level of gene expression can be quantified by measuring the emitted relative light units (RLU) with a luminometer. In this assay, IFN-α6 and IFN-α1 stood out as the strongest and weakest inducers of gene expression, respectively, over a large dose range (1 to 10,000 pg/ml) (Fig. 1A). All the other IFN-α subtypes did not differ significantly in their signaling activities for the doses tested compared to the results for IFN-α2. Notably, at higher concentrations, differences in reporter gene induction in response to the various IFN-α subtypes waned and were no longer visible as of 1,000 pg/ml, except for IFN-α1, which had to be administered at a dose greater than 1,000 pg/ml to achieve gene expression levels equivalent to those induced by IFN-α2. Although IFN-α6 displayed a superior signaling efficiency, the convergence of gene expression levels suggests the existence of a saturating effect in IFN-α signaling through the IFNAR complex, i.e., maximal receptor complex activation.

### IFN-α subtypes induce similar ISG response profiles at 1,000 pg/ml.

To investigate whether the different IFN-α subtypes at high doses trigger similar cellular responses irrespective of their ability to block HIV, we determined the ISG response to IFN-α subtypes 1, 2, 6, and 14 at 1,000 pg/ml in primary CD4^+^ T cells (cells from three separate donors were tested) using the quantitative real-time interferons and receptors RT^2^ PCR Profiler array from Qiagen. For this analysis, we focused on the subtypes that showed the greatest differences in signaling potencies compared to that of IFN-α2 (Fig. 1A), as well as that of IFN-α14, because of the current interest in this subtype for HIV therapy (16, 17, 26). Note that only the IFN-α-responsive genes (i.e., ISGs) included in the PCR array (i.e., a total of 25 genes) were considered in our analyses, but gene expression results for the full panel of 84 genes included in the PCR array are provided in Fig. S1 in the supplemental material. We found that the quality of the responses engendered by the different IFN-αs was similar when applied at a high dose, as essentially the same set of genes was induced by all subtypes (Fig. 1B). Specifically, all subtypes were able to induce a majority (72 to 80%) of the genes included in our analyses by ≥2-fold (Table 1). Only two genes were uniquely induced by ≥2-fold by IFN-α6 and -α14 (see genes highlighted blue in Table S1), and only one gene was downregulated by IFN-α1 (see gene highlighted yellow in Table S1), whereas it was marginally upregulated by the other three subtypes (average fold upregulation, 0.78). If higher thresholds of gene upregulation were considered, differences in the number of genes induced by each subtype were not significant. Quantitatively, the level of gene upregulation by each IFN-α subtype was consistent with the subtypes’ known affinities to IFNAR2, i.e., IFN-α6 and -α14 gave the strongest gene expression responses, followed by IFN-α2 and then IFN-α1 (Fig. 1C). Only 10/25 genes were induced to different levels in at least one pair of IFN responses (Table S2), and yet, it is noteworthy that all of these genes were induced ≥2-fold. Taken together, these data suggest that IFN-α1, -α2, -α6, and -α14 effectively have identical signaling properties but differ in their signaling efficiencies, which is in line with what we observed in the experiment whose results are shown in Fig. 1A.

**TABLE 1:** Average numbers of genes induced in CD4^+^ T cells upon treatment with saturating dose of selected IFN-α subtypes^*a*^

Level of upregulation	No. of genes induced at indicated level (avg across 3 donors) by IFN-α subtype:				*P* value^*b*^
	1	2	6	14	
≥2-fold	18	18	20	20	0.0417
≥3-fold	17	18	18	18	0.0625
≥5-fold	16	16	16	18	0.1667

a This analysis considers all 25 genes shown in Fig. 1B. The saturating dose used was 1,000 pg/ml.

b Significance testing was done using Friedman’s test (i.e., nonparametric RM one-way ANOVA).

10.1128/mSphere.00637-18.1 FIG S1 IFN-α1, -α2, -α6, and -α14 induce a similar pattern of gene expression of ISGs in PBMCs. Heat map showing the fold expression, compared to matched untreated controls, of all genes included in the interferons and receptors RT^2^ Profiler PCR array (Qiagen). Download FIG S1, TIF file, 0.6 MB. Copyright © 2019 Schlaepfer et al. 2019 Schlaepfer et al. This content is distributed under the terms of the Creative Commons Attribution 4.0 International license.

10.1128/mSphere.00637-18.5 TABLE S1 Fold gene expression induced by IFN-α stimulation compared to the expression in unstimulated matched controls. Values represent average gene expression responses from three independent donors. Genes highlighted in blue or yellow correspond to genes uniquely induced ≥2-fold by IFN-α6 and -α14 or uniquely downregulated by IFN-α1, respectively. Download Table S1, DOCX file, 0.06 MB. Copyright © 2019 Schlaepfer et al. 2019 Schlaepfer et al. This content is distributed under the terms of the Creative Commons Attribution 4.0 International license.

10.1128/mSphere.00637-18.6 TABLE S2 Repeated measures two-way ANOVAs of differentially induced genes in the human interferon RT^2^ Profiler PCR array. Download Table S2, DOCX file, 0.06 MB. Copyright © 2019 Schlaepfer et al. 2019 Schlaepfer et al. This content is distributed under the terms of the Creative Commons Attribution 4.0 International license.

Hierarchal cluster analysis also revealed that the individual donors were stronger determinants for the ISG responses than the specific IFN-α subtype used for stimulation (Fig. 1B). Namely, although the ISG responses induced by IFN-α6 and -α14 always clustered together for each donor, ultimately, the responses induced by the different IFN-α subtypes in a given donor were more similar to one another than those induced by the same IFN-α subtype in the three different donors.

### Dose-dependent inhibition of HIV by IFN-α subtypes.

To examine whether the dose-dependent differential signaling potencies between IFN-α subtypes are related to their antiviral activities, we explored the ability of IFN-α subtypes applied at a range of concentrations to inhibit HIV replication in primary cells. For these experiments, we chose IFN-α1, -α2, and -α6 as representatives of low-, intermediate-, and high-signaling-potency IFN-α family members, respectively. As the antiviral activity of IFN-αs may depend on the inoculum dose (29), we first assessed the anti-HIV activity of IFN-α2 in peripheral blood mononuclear cells (PBMCs) challenged with different doses of HIV. PBMCs from six healthy donors were pretreated with increasing concentrations of IFN-α2 and infected with HIV at multiplicities of infection (MOIs) in the range of 0.001 to 0.1 (Fig. S2). We found similar dose-inhibition curves at the two lower doses of HIV and a convergence of antiviral potencies for all MOIs when cells were treated with 1,000 pg/ml of IFN-α2. Notably, at lower concentrations (i.e., 1 to 100 pg/ml), IFN-α2 was more efficient in inhibiting HIV replication in the PBMCs infected with an MOI of 0.1 than in those infected with lower MOIs (average 50% inhibitory concentration [IC_50_] was 236.9 pg/ml at an MOI of 0.001, 96.7 pg/ml at an MOI of 0.01, and 14.5 pg/ml at an MOI of 0.1). Since we got vigorous HIV replication in all cell types/cultures tested at an MOI of 0.01 (Fig. S3) and we got dose-dependent inhibition of HIV replication with an MOI of either 0.1 or 0.01 even though they differed (Fig. S2), we used the lower MOI to economize on viral stocks.

10.1128/mSphere.00637-18.2 FIG S2 IFN-α2’s anti-HIV activity is only marginally affected by the MOI used to infect PBMCs. PBMCs from six donors were pretreated with different doses of IFN-α2 and infected overnight with HIV YU-2. The next day, the cultures were washed and IFN-α2 was added. Supernatants were harvested over the next 12 days, and p24 Ag concentrations quantified. Significance testing was done using RM two-way ANOVAs, comparing all groups to one another. Significant differences are indicated as follows: * , comparisons between MOIs of 0.1 and 0.01; # , comparisons between MOIs of 0.1 and 0.001. Levels of significance are indicated as follows: single symbols, *P* < 0.05; double symbols, *P* < 0.01; triple symbols, *P* < 0.001; quadruple symbols, *P* < 0.0001. IC_50_s were calculated using curve fitting (GraphPad Prism 7.04). Download FIG S2, TIF file, 0.8 MB. Copyright © 2019 Schlaepfer et al. 2019 Schlaepfer et al. This content is distributed under the terms of the Creative Commons Attribution 4.0 International license.

10.1128/mSphere.00637-18.3 FIG S3 IFN subtypes show clear dose-dependent HIV inhibition in primary cells. Isolated CD4^+^ T cells from 12 donors and MDMs and PBMCs from 9 donors each were pretreated at increasing doses with the different IFN subtypes and infected overnight with HIV YU-2. The next day, the cultures were washed, and the different IFN subtypes were added again. Supernatants were harvested over the next 11 to 12 days, and p24 Ag concentrations quantified. Download FIG S3, TIF file, 2.3 MB. Copyright © 2019 Schlaepfer et al. 2019 Schlaepfer et al. This content is distributed under the terms of the Creative Commons Attribution 4.0 International license.

**FIG 2:**
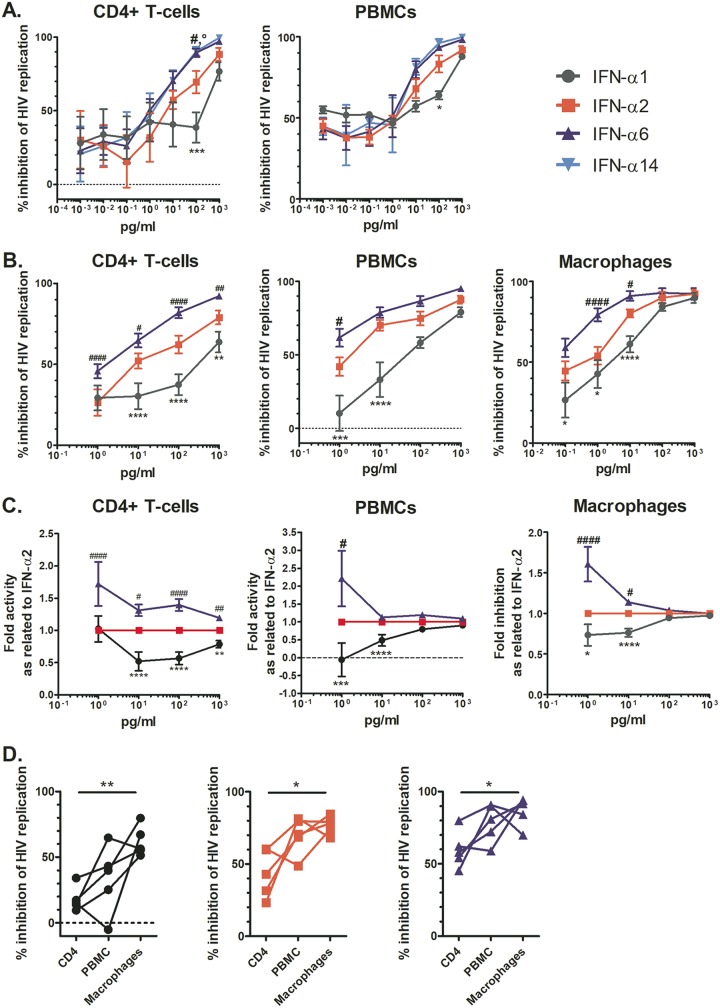
Dose-dependent HIV-inhibitory activity of IFN-α subtypes as examined in primary cells infected *ex vivo*. (A and B) CD4^+^ T cells, PBMCs, or MDMs were pretreated with the indicated IFN-α subtype for 2 h and then inoculated with HIV YU-2 overnight. The next day, the cultures were washed and the IFN-αs added back. At days 0, 4, 8, and 12, supernatant was harvested to monitor HIV replication by quantifying p24 Ag. The area under the curve of the p24 Ag over time was calculated and normalized to that of the control (i.e., no IFN-α). (A) Data exploring anti-HIV activities of IFN-α1, -α2, -α6, and -α14 in subsets of six donors’ CD4^+^ T cells and three donors’ PBMCs at an extended dose range. (B) Data for IFN-α1, -α2, and -α6 in cells from larger numbers of donors (12, 9, and 9 donors, respectively), focusing on higher dosages of the IFN-α subtypes. Responses were compared to those measured in the IFN-α2-treated groups by significance testing with RM two-way ANOVAs, comparing all groups to the IFN-α2 group. Symbols for comparisons with IFN-α2 are as follows: *, IFN-α1; #, IFN-α6. (C) Data for fold changes in anti-HIV activities compared to that of IFN-α2 for IFN-α subtypes and donor cells as described in the legend to panel B. (D) Percentages of inhibition in donor-matched cell subsets for IFN-α1, -α2, and -α6 at 10 pg/ml. Significance testing was done for each IFN-α subtype using RM one-way ANOVAs, comparing the responses in each cell type to one another.

In a first pilot experiment, we tested a wide range of IFN-α doses (0.001 to 1,000 pg/ml) and observed a clear dose-dependent inhibition of HIV replication in CD4^+^ T cells (*n* = 6) and PBMCs (*n* = 3) for all IFN-α subtypes tested as of a dose of 1 pg/ml (Fig. 2A). IFN-α6 and -α14 were more potent than IFN-α2 in inhibiting HIV, and IFN-α1 had the lowest potency, in line with the literature (16, 17); however, the anti-HIV activities of the subtypes studied converged at 1,000 pg/ml. Thus, we followed up with an extensive analysis of the anti-HIV activities of IFN-α subtypes 1, 2, and 6 administered at concentrations ranging from 1 to 1,000 pg/ml in primary cells from an additional six healthy donors (Fig. 2B and Fig. S3). We did not include IFN-α14, as it displayed anti-HIV potency highly comparable to that of IFN-α6 in the pilot experiment (by repeated measures [RM] two-way analysis of variance [ANOVA]: for CD4^+^ T cells, *P* = 0.9819, and for PBMCs, *P* = 0.5184). The results from this second screen corroborated the data from the pilot experiment, i.e., IFN-α6 was clearly the most potent anti-HIV IFN-α subtype, followed by IFN-α2 and then IFN-α1, in all cell types tested. We normalized the data by first calculating the area under the curve (AUC) of the p24 antigen (Ag) level over time for each condition and then expressing it as a percentage of that of the mock-treated HIV-infected control (Fig. 2B). We did this normalization because of the huge variability in the donors’ HIV permissiveness (at day 12, the mean p24 Ag levels [95% confidence interval], measured in pg/ml, were 49,152 [21,914 to 76,391] for HIV-infected CD4^+^ T cells, 17,686 [3,958 to 31,414] for HIV-infected monocyte-derived macrophages (MDMs), and 20,282 [1,359 to 39,206] for HIV-infected PBMCs), which might mask the actual anti-HIV effect when solely looking at p24 values. In any case, there is exactly the same trend when looking at the p24 Ag raw data (Fig. S3), a dose-dependent anti-HIV effect with IFN-α6 being the most potent IFN subtype and IFN-α1 being the least potent one.

When normalized to the anti-HIV activity of IFN-α2, the anti-HIV activities of the IFN-α subtypes examined converged at higher dosages (Fig. 2C), suggesting a saturation of antiviral activity as of 1,000 pg/ml of any IFN-α. Of note, the small differences in anti-HIV activity between subtypes that persisted in CD4^+^ T cells at 1,000 pg/ml suggest that this IFN dose is not sufficient to saturate the system in this cell type. The residual HIV replication (*V*_res_) observed is reminiscent of previous work examining the ability to inhibit founder HIV strains by either IFN-α2 or -β (30, 31). Moreover, cell type-specific differences in the HIV inhibitory activities of the various IFN-α subtypes tested were observed, with CD4^+^ T cells being overall less responsive to IFN-α treatment than PBMCs and MDMs (Fig. 2D). Furthermore, overall, we found that the anti-HIV responses in CD4^+^ T cells treated with IFN-α were stratified in terms of donor, with certain donors consistently displaying low (e.g., donor 577) or high (e.g., donor 597) responsiveness to IFN-α treatment regardless of the subtype (Fig. S4). The observed interdonor variability (50.24%, versus 36.86% attributed to IFN-α subtype; two-way ANOVA) supports the notion that some individuals are more responsive than others to IFN-α treatment (host factors associated with resistance to IFN-α treatment are reviewed in reference 32).

10.1128/mSphere.00637-18.4 FIG S4 Donors are stronger determinants of antiviral response to IFN-α treatment than the subtypes themselves. Inhibition of HIV replication in CD4+ T cells from the 12 donors treated with 1,000 pg/ml of IFN-α1, -α2, or -α6. Download FIG S4, TIF file, 1.0 MB. Copyright © 2019 Schlaepfer et al. 2019 Schlaepfer et al. This content is distributed under the terms of the Creative Commons Attribution 4.0 International license.

### Only modest differences in antiviral activities of human IFN-α subtypes at maximal treatment doses.

**FIG 3:**
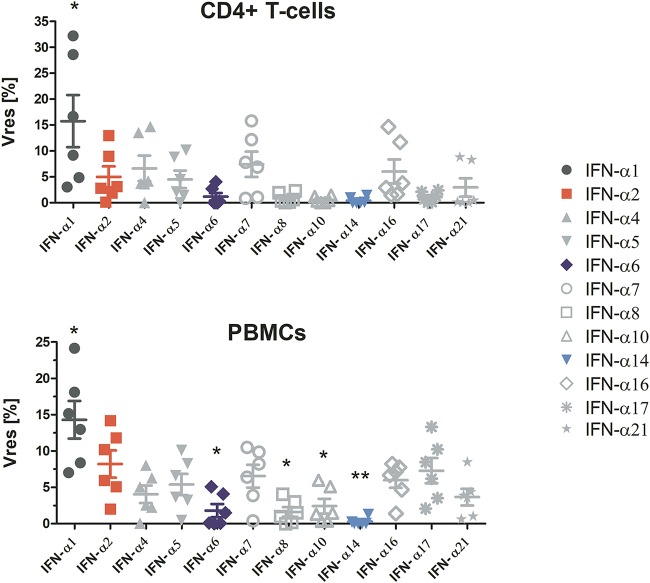
IFN subtypes converge in their anti-HIV activities at 1,000 pg/ml, with the exception of IFN-α1. PBMCs were pretreated with the IFN-α subtypes and infected with HIV YU-2 overnight. The next day, the cultures were washed and IFN-αs added back. *V*_res_ was assessed for all subtypes (*n* = 3 donors, in duplicates). Significance testing was done using RM one-way ANOVAs, comparing all IFN-α treatment groups to IFN-α2.

As we observed a convergence of the anti-HIV activities of IFN-α1, -α2, -α6, and -α14 at high treatment doses, we tested the anti-HIV effects of all IFN-α subtypes at 1,000 pg/ml in CD4^+^ T cells and PBMCs. This allowed us to evaluate the amount of residual HIV replication in the presence of maximally suppressive IFN-α concentrations compared to the level of viral replication in the untreated control (*V*_res_) (Fig. 3) (30). For all IFN-α subtypes, with the exception of IFN-α1, the mean *V*_res_ was below 10% HIV replication. These results mirror the dose-dependent trends in the IFN-αs’ signaling activities (Fig. 2) and point toward similar anti-HIV activities between IFN-α subtypes at high treatment doses. Note, we again observed cell type-specific differences in the *V*_res_ for the IFN-α subtypes.

### Preserved affinity of IFN mutants for IFNAR2 is critical for their anti-HIV activity, but reduced affinity of IFN mutants for IFNAR2 might be compensated by increased affinity for IFNAR1, resulting in superior anti-HIV activity.

**FIG 4:**
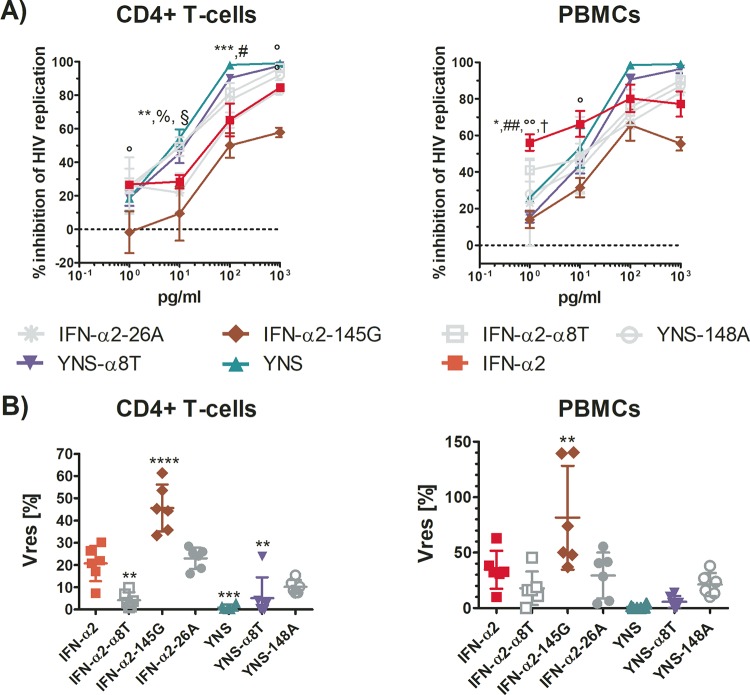
IFN-α2 mutants with modified IFNAR binding affinities display differential anti-HIV activities compared to that of IFN-α2. (A) Purified primary CD4^+^ T cells and PBMCs were pretreated with either IFN-α2 or the mutants for 2 h and then inoculated with HIV YU-2 overnight. The next day, the cultures were washed and IFN-αs added back. At days 0, 4, 8, and 12, supernatants were harvested for monitoring HIV replication by quantifying p24 Ag. The areas under the curve of the p24 Ag over time were calculated and normalized to that of matched controls (*n* = 3 donors). Significance testing was done using RM two-way ANOVAs, comparing all mutants to IFN-α2. Symbols for comparisons with IFN-α2 are as follows: *, YNS; #, YNS-α8T; §, YNS-148A; °, IFN-α2-145G; %, IFN-α2-α8T; †, IFN-α2-26A. (B) *V*_res_ values for all of the mutants (*n* = 3 donors, in duplicates). Significance testing was done using RM one-way ANOVAs, comparing mutants to IFN-α2.

Using a panel of IFN-α2 mutants with different affinities for IFNAR1 or -2, we examined the role of IFNAR1 versus that of IFNAR2 in inhibiting HIV replication in primary CD4^+^ T cells and PBMCs (Fig. 4). The binding affinities of the IFN-α2 mutants are given in relation to the binding affinity of IFN-α2 (set as 1), and an overview of these mutants is provided in Table 2. The IFN-α2 A145G variant (IFN-α2-145G; bearing a change of A to G at position 145) (33), which displays an ∼33-fold reduction in binding to IFNAR2, stood out for its marked reduction in anti-HIV activity compared to that of IFN-α2. Notably, the IFN-α2 L26A variant (IFN-α2-26A) (34), which binds to IFNAR2 with only ∼5-fold lower affinity than IFN-α2, exerted anti-HIV activity comparable to that of IFN-α2 (*P* = 0.9959). On the other hand, the YNS variant (bearing the mutations H57Y, E58N, and Q61S) (35), which has a binding affinity for IFNAR1 ∼60 times stronger than that of IFN-α2, displayed superior anti-HIV activity in CD4^+^ T cells (*P* = 0.0002) and a similar trend in PBMCs (*P* = 0.0559), pointing to the importance of IFNAR1 for the potency of the anti-HIV response. The YNS variant with the C-terminal tail of IFN-α8 (YNSα8T) (36), which exhibits a ∼22-fold increased binding affinity for IFNAR2 in addition to a high binding affinity for IFNAR1, showed anti-HIV activity comparable to that of YNS in CD4^+^ T cells (paired *t* test, *P* = 0.3488) and PBMCs (paired *t* test, *P* = 0.0921), suggesting that the additional increased binding affinity of this mutant for IFNAR2 did not augment its antiviral activity. Indeed, the IFN-α2 variant with only its C-terminal tail exchanged for that of IFN-α8 (IFN-α2-α8T) (34) showed antiviral activity comparable to that of IFN-α2 in PBMCs (*P* = 0.5982), whereas in CD4^+^ T cells, it surprisingly exerted a higher antiviral activity (*P* = 0.0015). Moreover, the YNS M148A variant (YNS-148A) (25), which displays ∼60-fold reduced binding to IFNAR2 and ∼60-fold increased binding to IFNAR1, blocked HIV replication similarly to IFN-α2 (*P* = 0.7823), suggesting that this mutant compensated for its reduced binding to IFNAR2 with its markedly higher binding to IFNAR1. Thus, binding to IFNAR2 appears to be critically needed for an effective anti-HIV response, but increased binding to IFNAR1 can potentiate antiviral potency or compensate for reduced binding to IFNAR2.

**TABLE 2:** Overview of IFN-α2 mutants used in this study

Mutant	Description of mutation(s)	Binding affinity for^*a*^:		Anti-HIV activity (*P* value)^*b*^	Reference(s)^*c*^
		IFNAR1	IFNAR2		
IFN-α2-145G	A145G	1	−33×	−24.94 (<0.0001)	33
IFN-α2-26A	L26A	1	−5×	−2.364 (0.9789)	34
IFN-α2-α8T	C-terminal tail replaced with that of IFN-α8	1	+20×	16.46 (0.0015)	34
YNS	H57Y E58N Q61S	+60×	1	19.68 (0.0002)	35
YNS-α8T	YNS + C-terminal tail replaced with that of IFN-α8	+60×	+20×	15.55 (0.0027)	36
YNS-148A	YNS + M148A	+60×	−60×	10.5 (0.0608)	1, 24, 25

a Binding affinity compared to that of IFN-α2.

b Mean difference in *V*_res_ results compared to the result for IFN-α2 in CD4^+^ T cells (see Fig. 4B). Significance testing was done using RM one-way ANOVAs, comparing all mutants to IFN-α2.

c The references cited in the last column are the papers that assessed the mutants’ binding affinities for IFNAR1 and IFNAR2.

## DISCUSSION

Given the prevailing controversy surrounding the functional role of the 12 distinct human IFN-α subtypes that all signal through a common receptor complex, in this study, we reexamined whether IFN-α subtypes induce different biological activities, with a focus on how the IFN-α treatment dose affects HIV replication. Whether and how IFN-α subtypes administered systemically at clinically relevant doses (i.e., supraphysiological doses) (19) may induce differential antiviral or antiproliferative activities is important to fully harness their therapeutic efficacy. Overall, our results support the idea that while IFN-α subtypes display different potencies in their various cellular activities, they do not intrinsically induce different biological responses, given that each subtype is exogenously applied at a high enough dose.

We show that differential levels of induction of ISRE-dependent gene expression by the 12 IFN-α subtypes are dose dependent, with differences only evident at lower treatment doses and a convergence of the signaling activities of all subtypes at treatment doses above 100 pg/ml, except for IFN-α1, which required doses above 1,000 pg/ml. Based on those data, the IFN subtypes can be categorized as low (IFN-α1), intermediate (IFN-αs 2, 4, 5, 7, 8, 10, 16, 17, and 21), or high (IFN-αs 6 and 14) inducers of gene expression at subsaturating doses, a classification which largely reflects the subtypes’ IFNAR2 binding affinities (7). This is in line with the concept that although full activation of the IFNAR occurs with different efficiencies for each subtype, once the receptor is fully activated, IFN signaling is indistinguishable between subtypes (27). The fact that IFN-α1’s binding affinity for IFNAR2 is approximately 2 orders of magnitude weaker than those of all other subtypes may explain why IFN-α1 appeared as an outlier and necessitated a much higher dose to reach maximal signaling activity. Furthermore, we demonstrate that disparities in the anti-HIV activities of IFN-α subtypes are also dose dependent (Fig. 2), with reduced antiviral activity compensated for by increasing the treatment dose. Notably, differences in the IFN-α subtypes’ dose-response curves provide evidence that the subtypes are unequally potent against HIV (IFN-α6 > IFN-α2 > IFN-α1), which, as discussed previously, is likely to be a consequence of differences in their affinities for IFNAR. Nevertheless, at maximal treatment concentrations, all IFN-α subtypes except IFN-α1 displayed similarly high anti-HIV effects (i.e., *V*_res_ of <10%) (Fig. 3), and we suppose that at even higher treatment doses, the maximal level of HIV inhibition achieved by each subtype would be indistinguishable. Thus, despite disparities in their potencies, our results suggest that IFN-αs intrinsically have comparable anti-HIV activities. These findings also highlight the importance of considering a wide range of IFN doses in order to be able to distinguish genuine differences in the functional activities of IFNs from dose-dependent differences in potencies and signaling efficiencies. Furthermore, macrophages were more responsive than CD4^+^ T cells to IFNs. This might be due to the higher expression level of IFNAR in monocytes/macrophages than in CD4^+^ T cells (37).

The dose-related effects identified in this study are not without precedence in the IFN body of literature (14, 27, 30, 38). For example, Moll et al. reported that the maximum ISG expression levels were ultimately comparable between IFN-α subtypes even though the subtypes displayed different potencies in terms of the dose required for half-maximal ISG induction. In their study, differences in IFN-α activities were only detected at subsaturating doses (14). Similarly, Fenton-May et al. showed that although exogenous IFN-α2 was more potent than IFN-β in inhibiting HIV replication (i.e., lower IC_50_ value), when maximally inhibitory concentrations of each subtype were applied, the extents of viral inhibition were similar (30). This dose effect may explain discrepancies between our findings and those of studies that, conversely, claim that IFN-α subtypes mediate differential anti-HIV activities (16, 17, 26, 28). Namely, Harper et al. reported differential antiviral activities between IFN-α subtypes, with IFN-α6, -α8, and -α14 blocking HIV most efficiently in *ex vivo*-infected human intestinal lamina propria aggregate cultures (17). In a follow-up study, Lavender et al. confirmed that IFN-α14 was more potent in suppressing HIV replication than IFN-α2 in a humanized mouse model (16), and these results were corroborated by Abraham et al., in whose study IFNs were compared in another humanized mouse model by hydrodynamic injection of plasmids encoding the IFNs (26). However, critically, in these three studies, only single treatment doses (i.e., 100 pg/ml or 1.5 × 10^5^ U/mouse) or unregulated IFN doses (i.e., expression from naked plasmid DNA) were investigated. Indeed, if we had only evaluated the level of HIV inhibition achieved with 100 pg/ml of IFN-α subtypes 1, 2, and 6, we would also have concluded that the subtypes mediated differential antiviral activities. However, in reality, the anti-HIV activities of these three proteins converged at higher doses. Therefore, although the aforementioned studies provide a valuable indication for including IFN-α14 in anti-HIV therapies, they do not provide evidence that IFN-α subtypes intrinsically differ in their functional roles.

Furthermore, we show that the ISG profiles induced in primary CD4^+^ T cells treated with a saturating dose (1,000 pg/ml) (Fig. 1A) of IFN-α1, -α2, -α6, and -α14 are qualitatively very similar (i.e., essentially identical sets of genes induced) (Fig. 1B). Nevertheless, differences in gene expression levels induced by the subtypes tested were observed (Fig. 1C and Table 1) and mirrored the subtypes’ binding affinities for IFNAR2, suggesting that binding affinity for IFNAR2 is an important determinant of the IFN-α subtypes’ signaling efficiencies and consequently, the level of ISG induction. We postulate that at higher treatment doses, these quantitative differences between subtypes would largely disappear, as seen in a study by da Silva et al., where IFN-α2 and IFN-β were capable of inducing qualitatively and quantitatively similar ISG profiles in human umbilical vein endothelial cells only when saturating doses of IFN were used (27). The similarity of the ISG responses produced by IFN-α1, -α2, -α6, and -α14 treatment is in accord with the aforementioned principle that the activated receptor complexes formed with IFNAR by the different IFN-α subtypes intrinsically possess the same signaling properties (albeit with different efficiencies) (27) and implies that sufficiently high IFN-α concentrations can compensate for the differences in signaling and gene expression. Contrarily, Cull et al. proposed that differential biological effects of IFN-α subtypes may be explained, at least in part, by their finding that IFN-α subtypes selectively activate different STAT molecules in J2E cells (38). However, in that study, only two IFN doses were tested in their STAT activation assays, limiting the validity of their claims, and even so, we can appreciate a dampening of differences in activation of STATs between IFNs at the higher dose.

Furthermore, we found that the cell donor had a greater influence on the ISG response profile and anti-HIV activity induced by IFN-α treatment than the actual subtype used for treatment (27). In other words, the genetic traits of the individual were dominant over more subtle biological differences induced by the different IFN-α subtypes. These results strongly support the notion of interdonor variability in responsiveness to IFN-α therapy (32) and highlight the importance of assessing whether a patient is likely to respond well to IFN-α therapy by evaluating known host factors involved in modulation of IFN-α responsiveness.

Finally, in an attempt to identify avenues by which IFN therapy could be rendered more effective, we used a panel of IFN-α2 mutants (Table 2) to identify patterns in binding affinities for IFNAR1 and IFNAR2 associated with differential anti-HIV potencies. The IFN-α2-145G mutant, which was the least potent IFN-α2 variant in our HIV inhibition dose dependency assays, is distinguishable from all other variants by its markedly reduced binding affinity for IFNAR2. On the other hand, the YNS mutant, which binds IFNAR1 with much greater affinity than IFN-α2, and the YNS-148A mutant, which in addition has a very low affinity for IFNAR2 compared to that of IFN-α2, both displayed significantly greater anti-HIV activities than IFN-α2 at maximal concentration. However, compared to one another, these two YNS mutants exerted comparable antiviral activities, implying that, provided there is strong IFNAR1 binding, binding to IFNAR2 is of lesser significance. Thus, we identify IFNAR2 binding as essential to IFN-α’s anti-HIV activity, whereas a heightened binding affinity for IFNAR1 enhances antiviral activity (Fig. 4). Notably, we show that in the case of reduced IFNAR2 binding, anti-HIV activity can be rescued by increased binding to IFNAR1.

In summary, we provide evidence that all the IFN-α subtypes exert potent anti-HIV activity and trigger a similar ISG response at saturating doses. Our investigations imply that the biological responses induced by IFN-α subtypes are intrinsically equivalent, with lower functional activities easily compensated for by increasing treatment doses. We propose that reported differences between IFN-α proteins largely arise from the use of subsaturating treatment doses and that, therefore, dose effects should always be considered in analyses of IFN-α subtypes’ functional activities. With regard to IFN-α therapy, our study identifies the development of mutants with increased binding affinity for IFNAR1 rather than the use of a specific IFN-α subtype as a promising avenue to improve treatment outcomes. Finally, it is imperative that interindividual differences in responsiveness to IFNs are considered in future studies investigating these proteins’ biological activities and potencies (16, 17, 26).

Of note, we identify some limitations to our study. First, we interrogated the biological activities of IFN-α subtypes in simplistic primary cell models. Although primary cells are more physiologically relevant systems than cell lines, they cannot fully recapitulate the complexity of the human immune system’s interaction with pathogens. The differences in response to IFN-α treatment between CD4^+^ T cells, PBMCs, and MDMs highlight this notion. Second, large variations in the responses to IFN-α treatment due to variability in donor responsiveness, as well as donor-dependent differences in HIV replication levels, reduced our ability to statistically validate significant trends in the data. To account for these sources of intergroup variability, we used repeated measures in our study design and analyses. A prescreening of donor host factors prior to use of that donor’s samples may be judicious in future studies. Third, we did not include all 12 IFN-α subtypes in all our assays, and thus, we cannot exclude the possibility that one of the subtypes not extensively tested displays some superior biological functionalities. Nevertheless, like many other studies, we extensively assessed the biological activities of representative IFN-α subtypes from each class of IFNAR binding affinities.

## MATERIALS AND METHODS

### Cells and reagents.

The human retinal pigment epithelial cell line containing an ISRE-driven reporter gene (ATCC CRL2302) was grown in Dulbecco modified Eagle medium (DMEM) supplemented with 10% fetal calf serum (FCS) and 1% penicillin–streptomycin. Peripheral blood mononuclear cells (PBMC) were isolated from buffy coats using Ficoll gradient centrifugation. CD4^+^ T cells were isolated using MicroBeads (Miltenyi). They were cultured in RPMI medium supplemented with 10% FBS, interleukin-2 (IL-2) at 10 U/ml, 2 mM l-glutamine, and 1% penicillin–streptomycin. Monocyte-derived macrophages (MDMs) were generated by culturing monocytes at a concentration of 5 × 10^4^ cells/well in a 96-well plate in RPMI 1640 medium supplemented with 10% human AB serum, 10 ng/ml macrophage colony-stimulating factor (M-CSF), 2 mM l-glutamine, and 1% penicillin–streptomycin for 6 days. Thereafter, cells were cultured for an additional 2 days in medium lacking cytokines.

The IFN-α subtypes were from PBL Assay Science (catalog number 11002-1). Furthermore, we examined a number of IFN-α mutants with different affinities, which were generated as previously described (35, 39).

### HIV infection.

The CCR5-tropic HIV YU-2 molecular clone was transfected into HEK293T cells using polyethyleneimine (Polysciences). Supernatants were harvested 48 h later, clarified by filtration (0.22 μM), aliquoted, and stored at −80°C until use. The titers of virus stocks were determined as described in reference 40. Briefly, 2 × 10^5^ CD8-depleted PBMCs from three healthy donors were activated with recombinant IL-2, polyhemagglutinin (PHA), and OKT3 antibody for 2 days and then challenged, in quadruplicate, with 1:5 serial dilutions of virus stock. After 7 days, supernatants were harvested and p24 antigen (Ag) concentrations were determined by an in-house p24 Ag enzyme-linked immunosorbent assay (ELISA). The 50% tissue culture infectious dose (TCID_50_) was then determined based on the number of p24-positive wells and the Reed and Muench calculation. For the HIV inhibition assays, 2 × 10^5^ PBMCs or CD4^+^ T cells were plated in triplicate in round-bottom 96-well plates, pretreated with the IFN-α subtypes for 2 h, and infected overnight with YU-2 at an MOI of 0.01, washed three times with phosphate-buffered saline (PBS), and then cultured in medium containing the different IFN-α subtypes. Culture supernatants were collected every 3 to 4 days and stored at −20°C until p24 Ag quantification by ELISA. The level of HIV inhibition (% HIV inhibition) was calculated by first determining the area under the curve (AUC) of the p24 Ag concentration over time, followed by expressing the AUC of the IFN-α-treated sample as a percentage of the matched mock-treated sample and subtracting this value from 100.

### Pathway-focused gene expression analysis using the interferons and receptors RT^2^ profiler PCR array.

We applied the interferons and receptors RT^2^ profiler PCR array (catalog number PAHS-064Z; Qiagen) protocol as provided by the manufacturer. Briefly, we stimulated 3 × 10^6^ PBMCs with IFN-α1, -α2, -α6, or -α14 at 1,000 pg/ml for 6 h. The cells were then harvested and lysed with the RLT lysis buffer. We then extracted RNA using the RNeasy minikit (catalog number 74106; Qiagen) and performed a DNase digest on the columns. Subsequently, we reverse transcribed 1 µg RNA for subsequent PCR on Bio-Rad iQ5. The gene expression analyses were done with Qiagen’s GeneGlobe data analysis software. Gene expression data for each IFN-α-treated sample are reported as fold changes relative to the results for matched untreated controls. Therefore, a value of zero signifies no increase in expression compared with the expression in the control samples. Since most of the genes in this PCR array are upregulated from slightly to very vigorously and only a few were modestly downregulated, we have chosen distinct scales for the up- versus downregulated genes. If we had applied the same scale for up- and downregulated genes, the downregulated ones would not be discernible.

### Statistical analysis.

All statistics were performed using the built-in analysis packages from GraphPad Prism 7.04 (GraphPad Software, Inc., La Jolla, CA). Data were analyzed with either repeated measures one-way or two-way ANOVA with Dunnett’s multiple-comparison test, unless stated otherwise in the text or in the figure legends. We considered a *P* value of ≤0.5 significant, and significance testing is reported as exact *P* values in the text or using symbols in the figures. Results are reported as mean values ± standard errors of the means (SEM), and figures depict group means and SEM, unless stated otherwise in the legend. Levels of significance are indicated as follows: single symbols, *P* ≤ 0.05; double symbols, *P* ≤ 0.01; triple symbols, *P* ≤ 0.001.
