# Supplementary figures and images for: Dose-Dependent Differences in HIV Inhibition by Different Interferon Alpha Subtypes While Having Overall Similar Biologic Effects
Source: mSphere. 2019 Feb 13;4(1):e00637-18. doi: 10.1128/mSphere.00637-18 (PMC6374594; doi:10.1128/mSphere.00637-18)

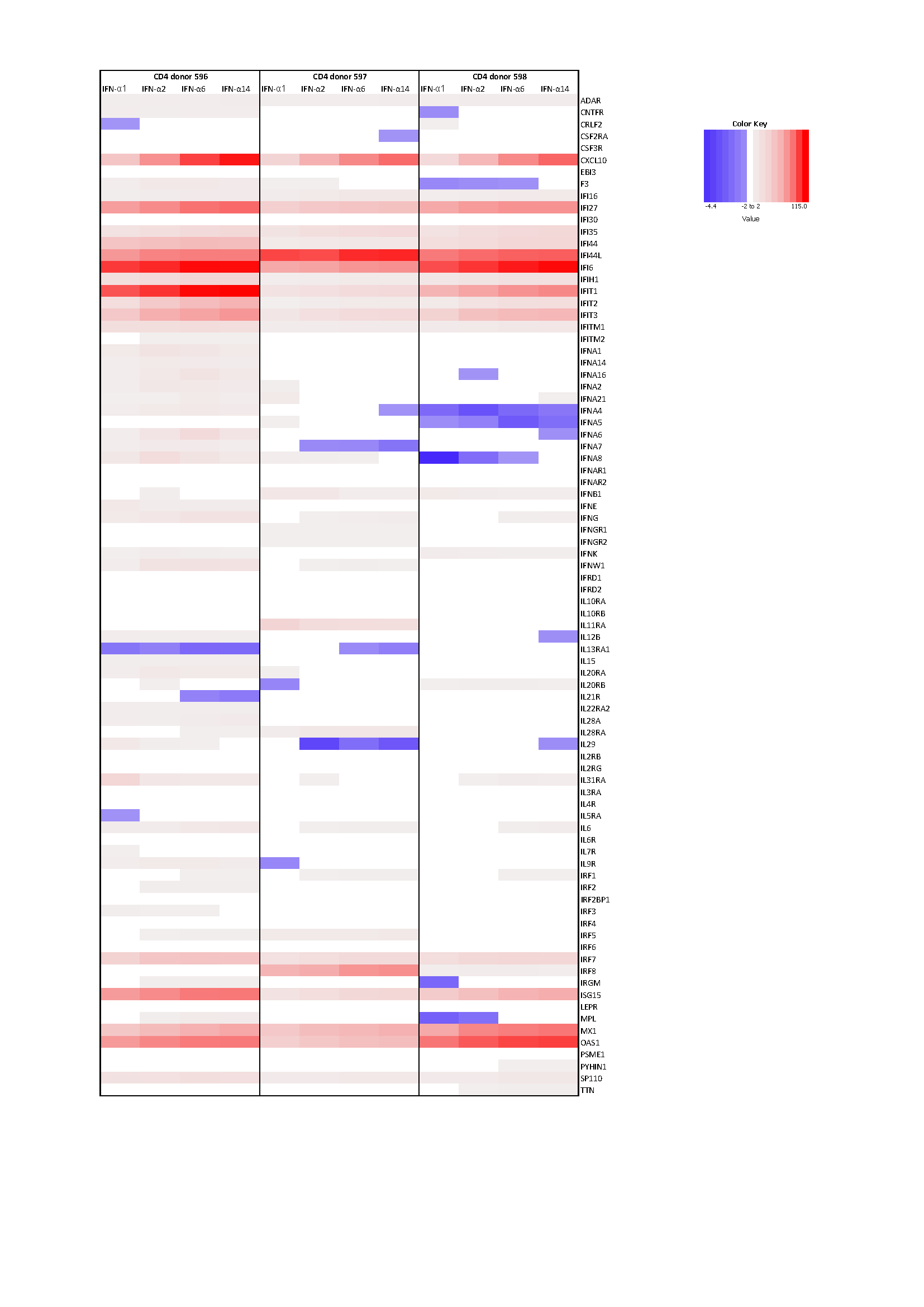

Supplement: FIG S1 [file mSphere.00637-18-sf001.tif]

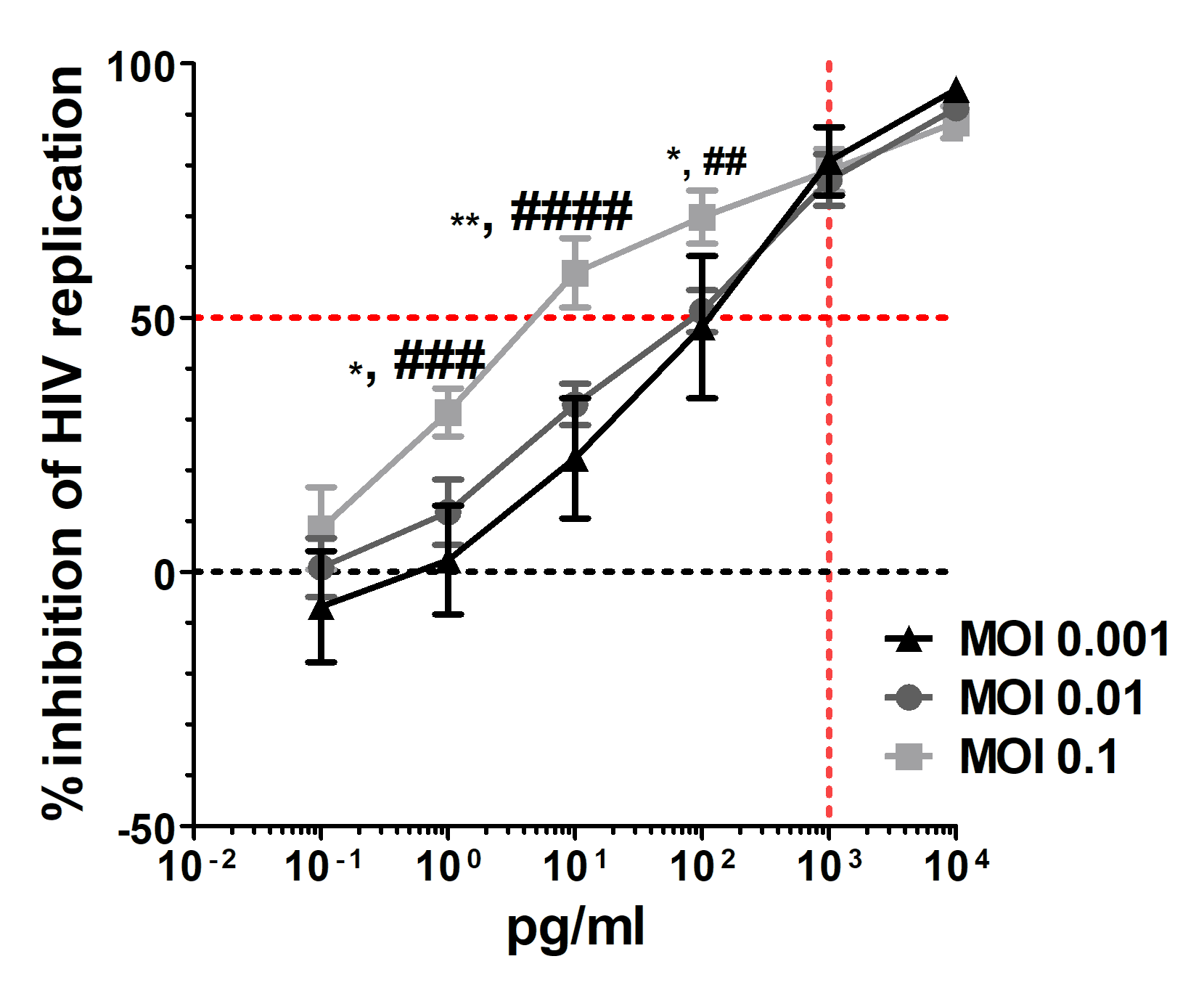

Supplement: FIG S2 [file mSphere.00637-18-sf002.tif]

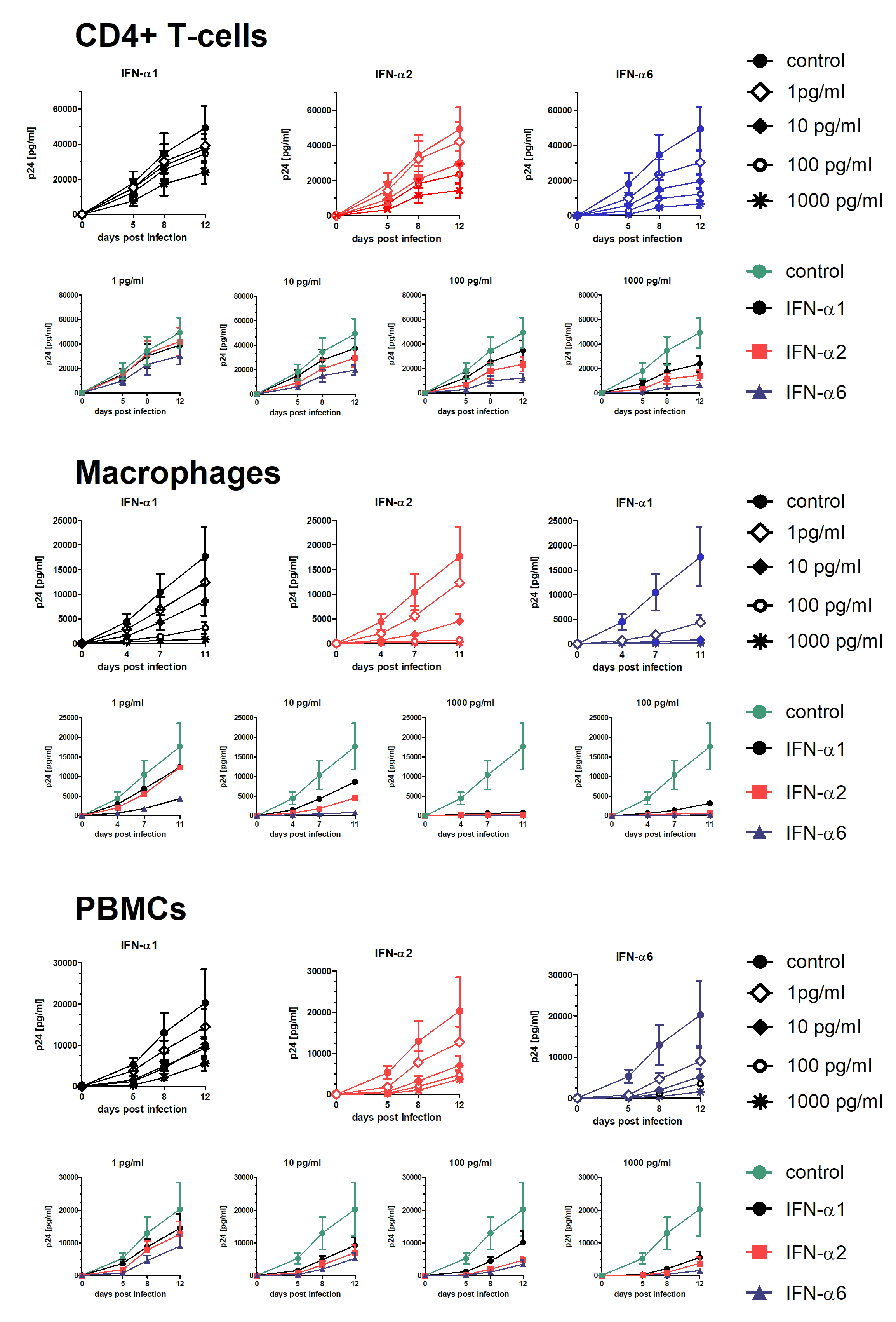

Supplement: FIG S3 [file mSphere.00637-18-sf003.tif]

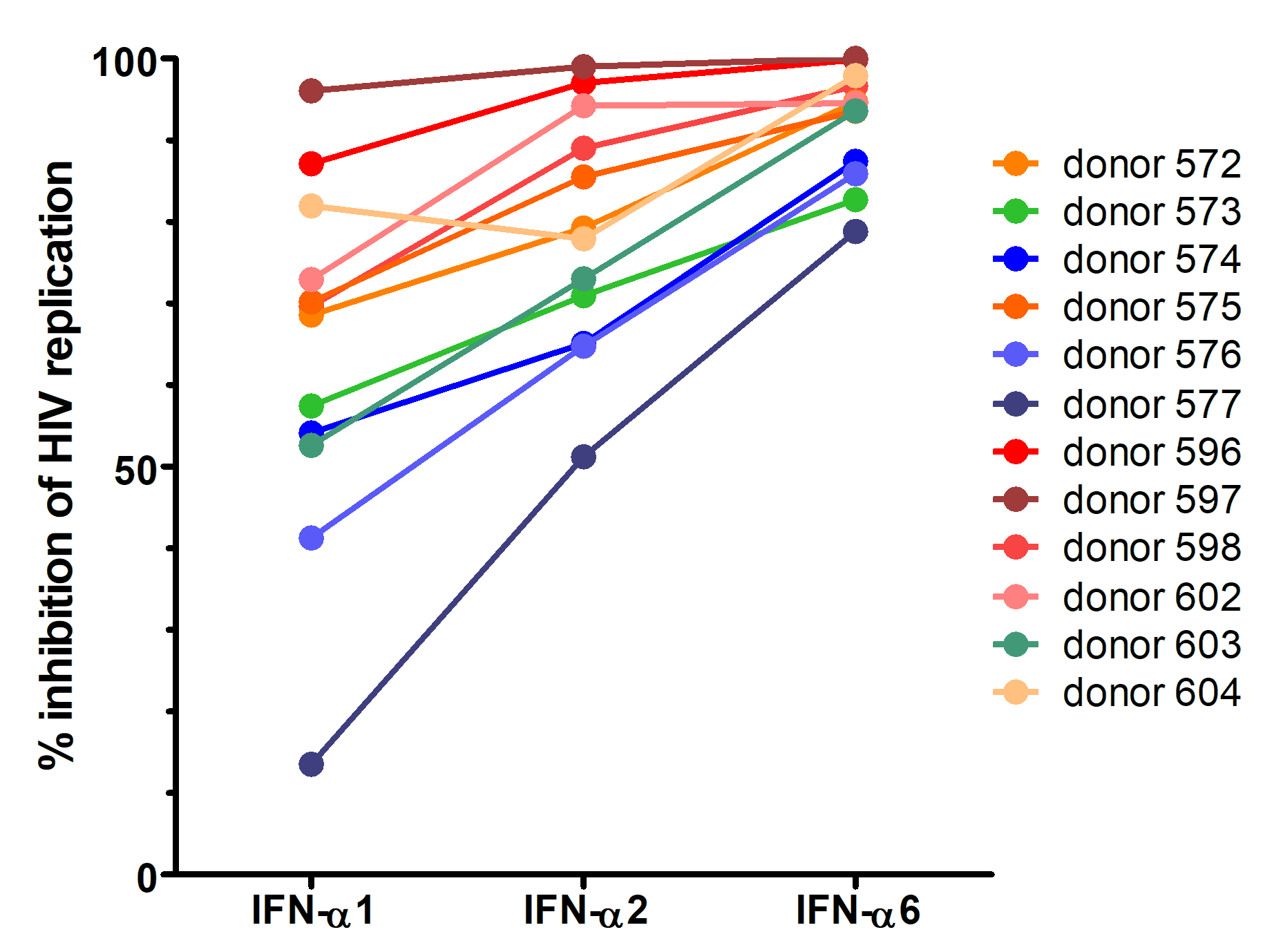

Supplement: FIG S4 [file mSphere.00637-18-sf004.tif]
