# Supplementary material for: Dose-Dependent Differences in HIV Inhibition by Different Interferon Alpha Subtypes While Having Overall Similar Biologic Effects
Source: mSphere. 2019 Feb 13;4(1):e00637-18. doi: 10.1128/mSphere.00637-18 (PMC6374594; doi:10.1128/mSphere.00637-18)
Supplement: TABLE S1 [file mSphere.00637-18-st001.docx]

**Table S1.**

|  | **Fold gene expression compared to unstimulated controls** | | | | | | | |
| --- | --- | --- | --- | --- | --- | --- | --- | --- |
|  |  | **IFN-α1** |  | **IFN-α2** |  | **IFN-α6** |  | **IFN-α14** |
| **ADAR** | 2.71 | | 3.18 | | 3.34 | | 3.38 | |
| **CXCL10** | 16.22 | | 35.37 | | 61.59 | | 79.16 | |
| **IFI16** | 3.14 | | 3.88 | | 4.46 | | 4.78 | |
| **IFI27** | 30.76 | | 37.10 | | 42.94 | | 44.82 | |
| **IFI30** | 0.57 | | 1.31 | | 1.36 | | 1.37 | |
| **IFI35** | 7.63 | | 10.04 | | 11.59 | | 12.53 | |
| **IFI44** | 12.42 | | 13.52 | | 14.99 | | 14.87 | |
| **IFI44L** | 61.14 | | 65.71 | | 73.45 | | 74.73 | |
| **IFI6** | 67.44 | | 74.91 | | 86.04 | | 89.17 | |
| **IFIH1** | 6.33 | | 7.71 | | 9.04 | | 9.42 | |
| **IFIT1** | 37.71 | | 45.70 | | 56.40 | | 59.32 | |
| **IFIT2** | 5.75 | | 10.31 | | 13.27 | | 15.13 | |
| **IFIT3** | 14.09 | | 21.70 | | 25.23 | | 28.26 | |
| **IFITM1** | 5.84 | | 5.83 | | 6.73 | | 6.63 | |
| **IFITM2** | 1.73 | | 1.78 | | 1.96 | | 1.97 | |
| **IRF1** | 1.59 | | 1.97 | | 2.19 | | 2.27 | |
| **IRF2** | 1.49 | | 1.86 | | 2.13 | | 2.17 | |
| **IRF7** | 11.46 | | 14.77 | | 16.04 | | 15.65 | |
| **IRGM** | -0.05 | | 0.77 | | 0.89 | | 0.70 | |
| **ISG15** | 22.85 | | 27.95 | | 33.45 | | 34.95 | |
| **MX1** | 25.30 | | 34.49 | | 38.40 | | 42.10 | |
| **OAS1** | 39.68 | | 48.27 | | 54.65 | | 56.16 | |
| **PSME1** | 1.52 | | 1.64 | | 1.72 | | 1.73 | |
| **PYHIN1** | 0.60 | | 1.38 | | 1.57 | | 1.62 | |
| **SP110** | 5.40 | | 5.65 | | 6.73 | | 6.60 | |
