# Supplementary material for: Dose-Dependent Differences in HIV Inhibition by Different Interferon Alpha Subtypes While Having Overall Similar Biologic Effects
Source: mSphere. 2019 Feb 13;4(1):e00637-18. doi: 10.1128/mSphere.00637-18 (PMC6374594; doi:10.1128/mSphere.00637-18)
Supplement: TABLE S2 [file mSphere.00637-18-st002.docx]

**Table S2.**

|  | IFN-α1 vs.  IFN-α2 | | IFN-α1 vs.  IFN-α6 | | IFN-α1 vs.  IFN-α14 | | IFN-α2 vs.  IFN-α6 | | IFN-α2 vs.  IFN-α14 | | IFN-α6 vs.  IFN-α14 | |
| --- | --- | --- | --- | --- | --- | --- | --- | --- | --- | --- | --- | --- |
|  | *Mean Diff.* | *P value* | *Mean Diff.* | *P value* | *Mean Diff.* | *P value* | *Mean Diff.* | *P value* | *Mean Diff.* | *P value* | *Mean Diff.* | *P value* |
| **CXCL10** | -19.15 | **<0.0001** | -45.38 | **<0.0001** | -62.94 | **<0.0001** | -26.22 | **<0.0001** | -43.79 | **<0.0001** | **-17.57** | **<0.0001** |
| **IFI27** | -6.333 | 0.0847 | -12.18 | **<0.0001** | -14.05 | **<0.0001** | -5.847 | 0.1278 | -7.717 | **0.0218** | **-1.87** | 0.8955 |
| **IFI44L** | -4.577 | 0.3157 | -12.32 | **<0.0001** | -13.59 | **<0.0001** | -7.74 | **0.0213** | -9.017 | **0.0048** | **-1.277** | 0.9633 |
| **IFI6** | -7.477 | **0.0281** | -18.6 | **<0.0001** | -21.73 | **<0.0001** | -11.13 | **0.0003** | -14.26 | **<0.0001** | **-3.13** | 0.6417 |
| **IFIT1** | -8 | **0.016** | -18.69 | **<0.0001** | -21.61 | **<0.0001** | -10.69 | **0.0005** | -13.61 | **<0.0001** | **-2.92** | 0.6908 |
| **IFIT2** | -4.57 | 0.317 | -7.52 | **0.0269** | -9.38 | **0.0031** | -2.95 | 0.6838 | -4.81 | 0.2726 | **-1.86** | 0.8969 |
| **IFIT3** | -7.61 | **0.0244** | -11.13 | **0.0003** | -14.17 | **<0.0001** | -3.523 | 0.5479 | -6.557 | 0.0693 | **-3.033** | 0.6644 |
| **ISG15** | -5.103 | 0.2238 | -10.6 | **0.0006** | -12.11 | **<0.0001** | -5.5 | 0.1678 | -7.003 | **0.0454** | **-1.503** | 0.9421 |
| **MX1** | -9.187 | **0.0039** | -13.1 | **<0.0001** | -16.8 | **<0.0001** | -3.917 | 0.4558 | -7.61 | **0.0244** | **-3.693** | 0.5076 |
| **OAS1** | -8.583 | **0.0082** | -14.97 | **<0.0001** | -16.48 | **<0.0001** | -6.387 | 0.0808 | -7.897 | **0.0179** | **-1.51** | 0.9414 |
